# Effects of almond consumption on sleep quality in adults: a randomized controlled trial

**DOI:** 10.3389/fnut.2026.1892442

**Published:** 2026-07-27

**Authors:** Sharvari R. Desai, Lancelot Pinto, Apurva Halbe, Rama A. Vaidya, Shobha A. Udipi, Soumik Kalita

**Affiliations:** 1 Nutrition, Kasturba Integrative Health Sciences - Medical Research Foundation, Mumbai, India; 2 Pulmonologist and Epidemiologist, P. D. Hinduja Hospital and Research Centre, Mumbai, India; 3 Endocrinology, Kasturba Integrative Health Sciences - Medical Research Foundation, Mumbai, India; 4 Family Physician (Lifestyle & Performance Medicine), FamPhy, Gurugram, India

**Keywords:** almonds, Epworth Sleepiness Scale (ESS), Pittsburgh Sleep Quality Index (PSQI), polysomnography, sleep quality, urban Indian adults

## Abstract

**Background:**

Sleep disturbances are associated with adverse metabolic, neurocognitive, and inflammatory outcomes. This study examined the effects of a 5-month whole-almond intervention on objective and subjective sleep parameters, biochemical and inflammatory markers in adults with poor sleep quality.

**Methods:**

In this open-label, parallel-arm, randomized controlled community-based trial with blinded outcome assessment, 175 adults aged 21–55 years with poor sleep quality (Pittsburgh Sleep Quality Index [PSQI] ≥ 5 and/or Epworth Sleepiness Scale [ESS] ≥ 10) were randomized to either an almond group (*n* = 88) or a control group receiving an isocaloric snack (*n* = 87) using computer-generated allocation. We hypothesized that regular almond consumption would improve subjective and objective sleep outcomes. Assessments conducted at baseline and after 5 months included polysomnography, PSQI, ESS, anthropometry, and biochemical markers. Per-protocol analyses were performed to evaluate between-group differences in mean changes.

**Results:**

Baseline characteristics were comparable between groups (*p* > 0.05). The almond group demonstrated significantly greater improvement in subjective sleep efficiency compared with controls (mean change: 9.7 ± 13.4 vs. 5.4 ± 10.6; *p* = 0.021). After adjustment for baseline values and sex, the almond group had lower global PSQI scores (3.94 ± 0.24 vs. 4.79 ± 0.32; *p* = 0.013), higher subjective sleep efficiency (94.45 ± 0.72% vs. 91.85 ± 0.42%; *p* = 0.012), and shorter subjective sleep latency (18.46 ± 2.25 vs. 26.29 ± 2.27 min; *p* = 0.016) than the control group. No significant treatment effects were observed for most PSG-derived sleep parameters, although wake during sleep tended to be lower in the almond group (*p* = 0.081). Endline melatonin concentrations were significantly higher following almond consumption (250.63 ± 15.98 vs. 206.19 ± 16.07 pg./mL, *p* = 0.050), whereas no significant differences were observed for other biochemical or anthropometric outcomes. A higher proportion of participants in the almond group achieved a clinically meaningful improvement in PSQI score and demonstrated reduced wake during sleep (*p* = 0.043).

**Conclusion:**

Almond consumption was associated with improvements in subjective sleep efficiency and sleep continuity in adults with poor sleep quality. These findings suggest that almonds may enhance perceived sleep quality, with slight objective improvements among responders.

**Clinical trial:**

https://ctri.nic.in/Clinicaltrials/showallp.php?mid1=80395&EncHid=&userName=Almond, Clinical Trials Registry–India (CTRI/2023/02/049727; registered 15 February 2023).

## 1 Introduction

Sleep is a fundamental biological process that sustains a wide spectrum of physiological and biochemical functions, including immune regulation, metabolic balance, appetite control, emotional resilience, and both endocrine and cardiovascular health (1, 2). Its role in enhancing physical and cognitive performance is well established. In recognition of its clinical relevance, the American Heart Association added sleep duration to its cardiovascular health metrics in 2022 (3). For adults, a minimum of 7 h of sleep per night is recommended to support optimal health (4, 5).

Duration alone does not determine sleep health; sleep quality is an important aspect of sleep. Sleep quality is assessed through parameters such as sleep latency, fragmentation, early awakenings, sleep efficiency, and sleep architecture, including slow-wave sleep (SWS) and rapid eye movement (REM) stages. These can be measured via subjective self-reports or objective tools such as polysomnography (6, 7).

Across populations, poor sleep quality and sleep disorders are emerging as major public health concerns. Prevalence estimates for insomnia, suboptimal sleep, and excessive daytime sleepiness range from 3.9 to 45.0% (8–10). In Indians, it has been reported that 57.2% of respondents in a COVID-era online survey experienced poor sleep quality, with an average sleep duration of 6.9 h and a sleep latency of 42.64 min (11). Among schoolchildren, 38.7% reported disturbed sleep, and over 50% slept less than 7 h per night (12). Adults with metabolic syndrome, particularly those with PCOS showed similar trends, with 57.9% sleeping less than 7 h daily (13). In an urban cohort of 758 Mumbai-based adults, nearly half (49.9%) reported poor sleep quality, and 30% experienced excessive daytime sleepiness (14). These findings point to a widespread and under-recognized sleep crisis in India.

Given the strong links between sleep health and chronic disease, there is growing interest in non-pharmacological strategies to improve sleep. These include behavioral interventions, sleep education, relaxation techniques, physical and mind–body exercises, aromatherapy, massage therapy, and psychotherapy (15). Among these, dietary approaches especially those rooted in traditional medicine and plant-based nutrition hold promising potential (16, 17).

Emerging evidence suggests that plant foods rich in unsaturated fats and sleep-supportive compounds may enhance sleep quality by modulating melatonin and serotonin pathways (18). Almonds, a nutrient-dense tree nut, contain mono and polyunsaturated fatty acids along with 209 mg/100 g of tryptophan, a precursor of serotonin and melatonin. They also contain approximately 39 ng of melatonin per g dry weight (19, 20). A 28 g serving of almonds delivers 7.3 mg of vitamin E, 77 mg of magnesium, and 208 mg of potassium (21), micronutrients known to support sleep via muscle relaxation and neuroendocrine regulation (22). Notably, it was observed that the severity of insomnia reduced among the medical students following a two-week regimen of 10 almonds daily (23). Almonds, have a strong biochemical rationale due to their nutrient profile, and small observational or short-term studies have indicated potential benefits for sleep. However, evidence from well-designed, long-duration randomized controlled trials especially those incorporating objective measures like polysomnography and biological markers has remained limited. To address this gap, the present randomized controlled trial was designed with an objective to assess the impact of daily almond consumption for 5 months on both subjective and polysomnography-based measures of sleep quality in 21–55-year-old urban Indian adults with poor sleep.

## 2 Methods

### 2.1 Study design and participants

This randomized, controlled, open-label, parallel-arm trial was conducted among community-dwelling young and middle-aged adults (21–55 years) residing in the Mumbai Metropolitan Region, India. The study period spanned March 2023 to January 2025. Ethical approval was obtained from the Intersystem Biomedical Ethics Committee, Mumbai (ISBEC approval dated 21 October 2022), and the trial was prospectively registered with the Clinical Trials Registry of India (CTRI/2023/02/049727) (24). Participants were recruited primarily from the community, including students, faculty members of academic institutions, and corporate employees in Mumbai. Written informed consent was obtained at two stages: before screening and again prior to enrolment in the intervention phase.

The study comprised two sequential phases. In the first phase, participants underwent screening to assess sleep quality and daytime sleepiness. Sleep quality was evaluated using the Pittsburgh Sleep Quality Index (PSQI), which assesses seven domains: subjective sleep quality, sleep latency, sleep duration, habitual sleep efficiency, sleep disturbances, use of sleep medication, and daytime dysfunction. The Epworth Sleepiness Scale (ESS) was used as a complementary tool to quantify daytime somnolence and the likelihood of falling asleep in routine situations.

Individuals with poor sleep quality, defined as a PSQI score >5 (25–27) and/or an ESS score >10 (28), and those reporting stable sleep habits over the preceding 6 months, were eligible and included in the study. Exclusion criteria included those participants reporting major sleep disorders (such as chronic insomnia, obstructive sleep apnea with apnea-hypopnea index >30, restless leg syndrome, narcolepsy, central apnea, or parasomnias), night-shift or irregular work schedules, regular use of sleep medications, melatonin, or sedatives, and known allergies to almonds. Participants with psychiatric or neurological conditions (including major depression, anxiety disorders, eating disorders, cognitive impairment, history of stroke, or substance abuse), those following prescribed diets in the preceding 6 months, individuals with chronic pain managed with sedatives or hypnotics, and active smokers or users of recreational substances were also excluded. Pregnant or lactating women, as well as women with children under 2 years of age who shared the sleeping environment, were not eligible for participation.

### 2.2 Intervention protocol

Eligible participants (PSQI score >5 and/or an ESS score >10) were randomized in a 1:1 ratio to either the almond intervention group or the control group after obtaining their consent to participate in the intervention trial. Randomization was conducted using computer-generated permuted blocks of variable sizes by an independent statistician. Allocation was concealed using sequentially numbered opaque sealed pouches. These eligible participants underwent overnight polysomnography (PSG) at baseline and at the end of the study.

Those participants who were eligible but diagnosed with severe obstructive sleep apnea (apnea–hypopnea index [AHI] > 30) after undergoing polysomnography at baseline were excluded from the intervention trial and advised to seek immediate consultation with a sleep specialist. We hypothesized that daily almond consumption over a 5-month intervention period would improve both subjective and objective sleep quality compared with an isocaloric control snack.

#### 2.2.1 Intervention

Participants in the experimental group received 60 g/day of raw almonds (approximately 50 whole almonds), provided by the Almond Board of California (United States), for 5 months. Almonds were supplied in two daily portions in resealable pouches and were to be consumed as a mid-morning snack and an evening snack. Storage conditions were standardized at 10–15 °C, and the products were delivered in hygienic cartons.

The control group received isocaloric Indian savory snacks (approximately 61–65 g/day; 350–360 kcal), prepared to match the caloric and protein value of 60 g of almonds. Examples included *khakra*, *chivda*, cookies, and crackers. Two servings were provided daily, rotated across seven varieties to minimize taste fatigue. Snacks were packaged in sealed laminated pouches. Supplies for both groups were distributed every week, with instructions to maintain usual dietary and lifestyle habits.

Participants in both groups were instructed to avoid consuming any other nuts or nut-based products throughout the 5-month intervention. They were also advised to avoid the regular consumption of caffeine-containing beverages and alcohol and to maintain their usual diet, physical activity, and daily routine throughout the study period.

#### 2.2.2 Compliance and monitoring

Adherence was assessed through weekly supervision, including in-person and video-call monitoring. Participants returned unused food portions twice weekly for weighing and intake estimation. Adherence was defined as consumption of at least 80% of the assigned product during the 20-week intervention. Serum alpha-tocopherol (Vitamin E) concentrations were measured at baseline and at the end of the study as an objective biomarker of compliance, given that almonds are a natural source of vitamin E. No serious adverse events or intervention-related harms were reported during the study period in either group.

Due to the dietary nature of the intervention and the fact that participants were recruited from the same community, participant blinding was not feasible. Therefore, this study was conducted as an open-label randomized controlled trial with blinded outcome assessment. However, investigators involved in outcome evaluation, polysomnography (PSG) technicians, laboratory personnel, and data analysts remained blinded to group allocation throughout the study to minimize assessment and analytical bias.

### 2.3 Sample size estimation

**Figure 1:**
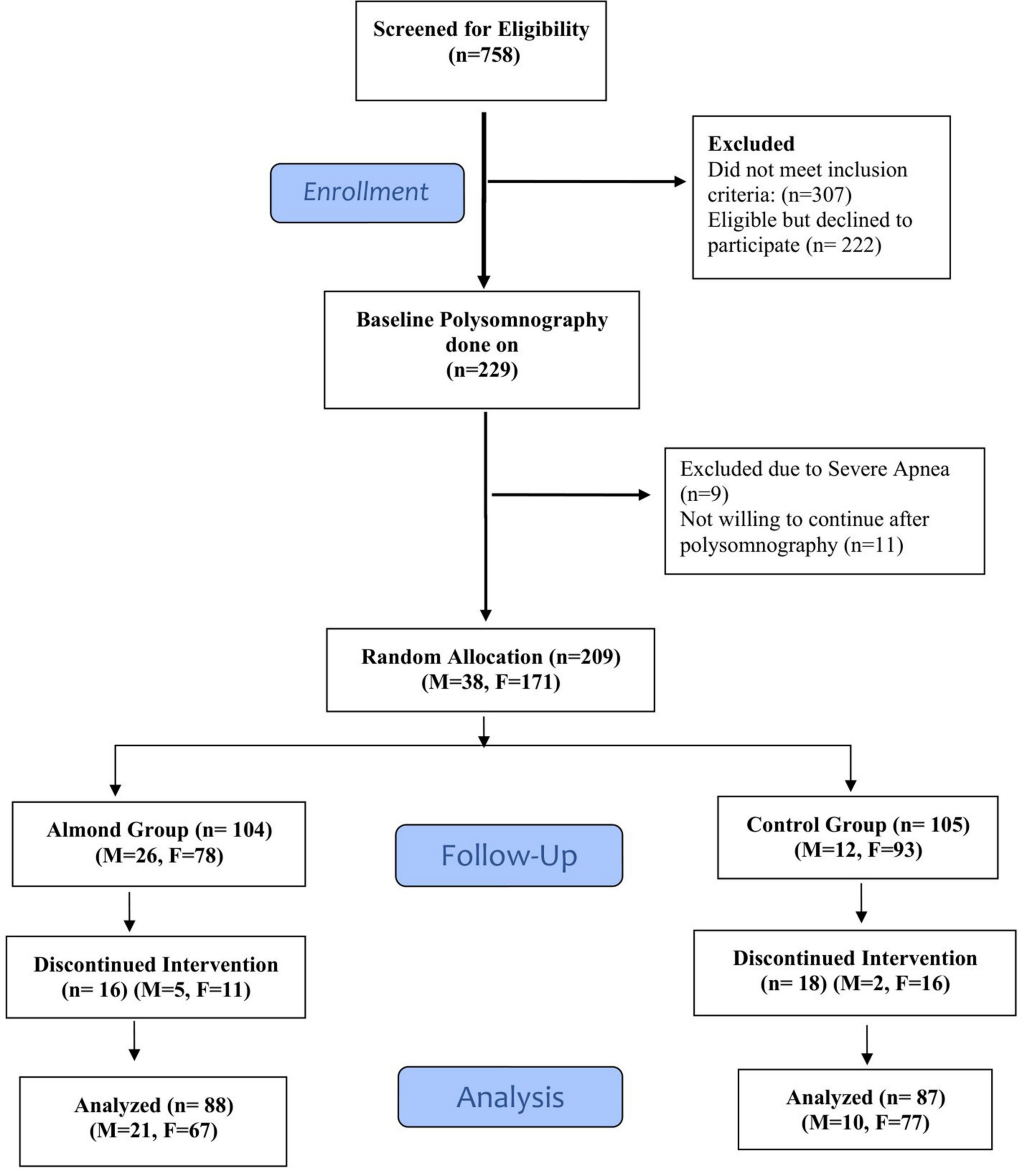
CONSORT diagram of participant screening, randomization, and final analysis.

Sample size determination was carried out using a two-sample means test (Z-test) with 95% confidence level and 80% power, the minimum required sample was 172 participants (86 per arm). Factoring in a projected attrition rate of 20%, the final sample size was expanded to 208 participants (104 per arm). A total of 758 adults were screened to arrive at the requisite sample size. Figure 1 presents the CONSORT flow diagram.

### 2.4 Assessments

All the assessments were done at the baseline and at the end of 5-months intervention.

#### 2.4.1 Polysomnography

Participants deemed eligible for inclusion by the study physician underwent Level 2 home-based polysomnography (PSG) using the Philips Alice PDX system. PSG assessments were conducted at baseline and following the 5-month intervention by certified polysomnographic technologists (Genotronics Pvt. Ltd. and Pulmonix Pvt. Ltd.), who were blinded to treatment allocation. Sleep recordings included electroencephalography (EEG) along with other standard polysomnographic parameters. A single overnight PSG recording was obtained at each assessment, with scheduling based on participants’ convenience to enhance ecological validity while minimizing participant burden. To account for potential differences in sleep patterns between weekdays and weekends, all PSG assessments were scheduled on weekdays. Follow-up PSG recordings were likewise conducted on a weekday, although not necessarily on the same day of the week as the baseline assessment.

Participants were instructed to finish dinner before 8:00 p.m. and were instructed to avoid having any caffeine-containing beverages and foods after 2:00 p.m. (or for at least 8 h before bedtime) on the day of polysomnography assessment. They were also asked to refrain from applying lotions, colognes, gels, or makeup prior to sleep. A certified polysomnography technologist visited participants’ residences between 9:00 p.m. and 10:00 p.m. for equipment setup. This time was selected as it was the usual time that most participants went to bed. Two dedicated certified PSG technologists worked with the research team and did the polysomnography. The same recording procedures and quality assurance protocol were applied to all participants to ensure consistency and reliability of the polysomnography recordings. Participants residing in nearby localities were scheduled on the same night, allowing each technician to perform home visits for not more than two participants on the same night. Technologists were assigned to each participant and performed the test at baseline and end of the study. Both technicians followed an identical standardized protocol for equipment setup, sensor placement, participant instructions, and device retrieval the following morning. The entire protocol was reviewed and field-tested on some of the research team members before the study commenced. It was made sure that polysomnography electrodes were placed on key anatomical sites: scalp, chin, temples, chest, and legs, using mild adhesive in the presence of an adult family member. The system included flexible wiring to allow movement and pulse oximetry via a clip on the middle finger. After waking in the morning, participants removed electrodes as instructed. Equipment was retrieved the following morning, and sleep scoring and staging were performed manually by the PSG technologist according to the American Academy of Sleep Medicine (AASM) scoring criteria. All polysomnography recordings and scoring reports were subsequently reviewed and verified by a certified sleep expert.

#### 2.4.2 Pittsburgh sleep quality index (PSQI)

Each participant was interviewed and the first 19 items were filled. The 19 self-reported items were grouped into seven distinct domains (25, 26):

- Subjective Sleep Quality: Overall perception of sleep quality.
- Sleep Latency: Time required to fall asleep.
- Sleep Duration: Total hours of actual sleep per night.
- Sleep Efficiency: Percentage of time spent in bed to percentage of time that is actually spent sleeping.
- Sleep Disturbances: Sleep disruptions such as nocturnal awakenings, bad dreams, or pain.
- Use of Sleep Medication: Frequency of use of prescription or over-the-counter sleep aids.
- Daytime Dysfunction: Frequency of sleepiness or difficulty remaining awake during daytime activities (e.g., driving or eating).

Each of the seven components was scored from 0 to 3, where (0) indicated no difficulty and (3) indicated severe difficulty. The global score ranges from 0 to 21, with higher score reflecting poor sleep quality. A global score >5 was used to identify poor sleepers, consistent with established sensitivity and specificity across diverse populations (25–27, 29).

#### 2.4.3 Epworth Sleepiness Scale (ESS)

The ESS is an eight-item instrument that assesses daytime sleepiness. Each item is scored from 0 (no chance of dozing) to 3 (high chance), yielding a total possible score of 24. An ESS score >10 was considered as a cut-off to predict daytime sleepiness (28). Interviews were conducted by trained investigators.

The PSQI and ESS questionnaires were administered during weekday study visits (Monday to Friday). Participants were interviewed and responses were based on their usual sleep patterns during the respective recall periods. The PSQI assessed habitual sleep quality over the preceding 1 month, whereas the ESS evaluated typical daytime sleepiness during routine daily situations.

#### 2.4.4 Anthropometry and body composition

Height was measured using a stadiometer, weight and body composition was done using InBody Model 120. Waist and hip circumference were measured using a calibrated SECA measuring tape. Body Mass Index (BMI) and waist-to-hip ratio were subsequently calculated. All measurements were obtained according to the standardized protocols, with participants wearing light clothing and no footwear.

#### 2.4.5 Biological samples and biochemistry

Following PSG confirmation of eligibility, participants underwent home-based venous blood collection by trained phlebotomists after an overnight fast of at least 12 h. Blood samples were collected at approximately 8:00 a.m. to minimize the effects of diurnal variation.

Serum glucose, insulin, glycated hemoglobin (HbA1c), and cortisol concentrations were analyzed at Agilus Diagnostics, Mumbai, an NABL-accredited clinical laboratory, using validated standard laboratory methods. Fasting plasma glucose was measured using the enzymatic hexokinase method, serum insulin by chemiluminescent microparticle immunoassay (CMIA), HbA1c by high-performance liquid chromatography (HPLC), and serum cortisol by immunoassay. Insulin resistance was estimated using the Homeostatic Model Assessment of Insulin Resistance (HOMA-IR), calculated as: HOMA-IR = [fasting serum insulin (μU/mL) × fasting plasma glucose (mg/dL)]/405 (30).

Serum concentrations of melatonin, serotonin, brain-derived neurotrophic factor (BDNF), interleukin-6 (IL-6), and tumor necrosis factor-alpha (TNF-*α*) were quantified using commercially available enzyme-linked immunosorbent assay (ELISA) kits (Elabscience Biotechnology Inc., Houston, TX, United States) according to the manufacturer’s instructions. Serum serotonin was quantified using the Human Serotonin (5-HT) ELISA Kit (Catalog No. E-EL-0033), brain-derived neurotrophic factor (BDNF) using the Human BDNF ELISA Kit (Catalog No. E-EL-H0010), tumor necrosis factor-alpha (TNF-α) using the Human TNF-α ELISA Kit (Catalog No. E-EL-H0109), melatonin using the Human Melatonin (MT) ELISA Kit (Catalog No. E-EL-H2016), and interleukin-6 (IL-6) using the Human IL-6 ELISA Kit (Catalog No. E-EL-H6156). All ELISA assays were performed according to the manufacturer’s instructions. The assay detection limits, measurement ranges, and intra-assay and inter-assay coefficients of variation are provided in Supplementary Table 1. Serum vitamin E was quantified by LC/MS–MS method using Sigma-Aldrich reference standards. All markers were assessed at baseline and repeated following a 5-month intervention.

### 2.5 Outcomes

The primary outcomes were improvement in sleep efficiency (measured by PSG and PSQI) and PSQI total score.

Secondary outcomes included changes in other PSG-derived sleep parameters (sleep duration, sleep latency, wake after sleep onset, REM sleep), anthropometric and body composition measures, and selected biochemical parameters.

### 2.6 Statistical analyses

All statistical analyses were performed using Statistical Package for Social Sciences, SPSS (Version 27). Per-protocol analysis was performed because only participants completing the intervention and endpoint assessments were included. Missing outcome data were not imputed. Attrition rates were comparable between groups, minimizing risk of attrition bias.

Continuous variables were expressed as mean ± standard deviation (SD), and categorical variables as frequencies and percentages. Changes from baseline to endline (Δ values) were calculated for all outcomes. Between-group differences in mean change were assessed using independent sample t-tests for normally distributed variables. A two-sided *p*-value < 0.05 was considered statistically significant. To evaluate intervention effects over time, a two-way repeated-measures analysis of variance (ANOVA) was performed with time (baseline and endline) as the within-subject factor and group (experimental and control) as the between-subject factor. To account for the unequal sex distribution between groups and potential sex-related differences in sleep outcomes, body composition, and biochemical parameters, sensitivity analyses were conducted using analysis of covariance (ANCOVA). Post-intervention values were entered as dependent variables, treatment group as the fixed factor, and baseline values together with sex as covariates. Further, participants achieving a ≥ 3-point reduction in PSQI score were classified as responders (31). Among these responders, between-group comparison was performed using independent t-tests for sleep parameters measured by polysomnography. This analysis evaluated whether intervention effects were more pronounced in clinically improved individuals. A *p* value < 0.05 was considered statistically significant.

## 3 Results

### 3.1 Participant characteristics

A total of 758 individuals were screened for eligibility. Of these, 451 participants (96 males and 355 females) met the inclusion criteria, defined as poor sleep quality indicated by a PSQI score >5 and/or an ESS score >10. Among these eligible individuals, 222 declined to provide consent for further participation, including undergoing polysomnography (PSG). Thus, 229 participants proceeded to the baseline PSG assessment.

Following PSG, 9 participants were excluded due to severe obstructive sleep apnea (AHI > 30), which met the study’s predefined exclusion criterion. An additional 11 participants withdrew voluntarily at this stage. Consequently, 209 participants were randomized using a computer-generated algorithm into either the almond group (*n* = 104) or the control group (*n* = 105). During the 20-week intervention, 34 participants were lost to follow-up. A total of 175 participants (83.7% of those randomized) completed the study and were included in the per-protocol analysis (almond group: *n* = 88; control group: *n* = 87), as illustrated in Figure 1.

#### 3.1.1 Adherence and compliance

All 175 participants who completed the trial met the predefined adherence criterion of consuming more than 80% of the assigned intervention food. Attrition rates were comparable between the almond and control groups, suggesting no intervention-related differential dropout. Compliance was reinforced through weekly monitoring, including follow-up calls and verification of returned unused portions. Biochemical evidence of adherence was demonstrated by a greater increase in serum alpha-tocopherol concentrations at endline in the almond group, consistent with consumption of vitamin-E–rich almonds.

#### 3.1.2 Baseline characteristics

Baseline demographic and clinical characteristics were similar between the almond group and control group. There were no statistically significant differences in age (39.5 ± 10.6 years vs. 39.3 ± 10.5 years, *p* = 0.900), BMI (27.03 ± 4.42 kg/m^2^ vs. 27.72 ± 5.10 kg/m^2^, *p* = 0.344), or global PSQI score (7.44 ± 2.75 vs. 7.57 ± 2.74, *p* = 0.752) between the almond and control group (Table 1).

**Table 1:** Baseline characteristics in almond and control groups.

Baseline participant characteristics	Almond group (*n* = 88)	Control group (*n* = 87)	*p*
Sex *n*(%)			
Male	21(23.9%)	15(17.2%)	0.062
Female	67(76.1%)	74(85.1%)	
Occupation *n*(%)			
Service	37(42%)	38(43.7%)	0.512
Business	2(2.3%)	3(3.4%)	
Housewives	25(28.5%)	31(35.6%)	
Professionals	24(27.3%)	15(17.2%)	
Age (in years), mean (SD)	39.5(10.6)	39.3(10.5)	0.900
BMI (kg/m^2^), mean (SD)	27.03(4.42)	27.72 (5.10)	0.344
PSQI Score, mean (SD)	7.44(2.75)	7.57(2.74)	0.752
ESS, mean (SD)	9.28 (3.91)	10.41 (4.55)	0.080
Sleep efficiency (%), mean (SD)	84.6(7.1)	83.9(5.5)	0.498

PSQI, Pittsburgh Sleep Quality Index; ESS, Epworth Sleepiness Scale. Independent *t*-test, *p* < 0.05 was considered significant.

Sleep-related measures, including ESS scores (9.28 ± 3.91 vs. 10.41 ± 4.55, *p* = 0.083) and sleep efficiency (84.6 ± 7.1% vs. 83.9 ± 5.5%, *p* = 0.498), were also comparable at baseline. Additionally, there were no significant differences in sex distribution or occupational categories (all *p* > 0.05), indicating strong baseline equivalence between groups (Table 1).

### 3.2 Changes in objective and subjective sleep parameters following the 5-month intervention

When within group changes were measured after 20 weeks, both the almond and control group demonstrated improvements in subjective sleep outcomes which included global PSQI score, sleep efficiency, sleep latency, sleep duration, and ESS score (all *p* < 0.001). Among objective sleep parameters, the almond group showed significant reductions in wake during sleep (31.46 ± 28.16 to 26.69 ± 22.52 min; *p* = 0.043), increased N3 sleep duration (154.30 ± 62.35 to 170.37 ± 72.15 min; *p* = 0.049), and reduced REM sleep duration (45.87 ± 25.05 to 38.57 ± 26.48 min; *p* = 0.003). In the control group, significant decreases in N2 sleep duration and REM sleep duration and an increase in N3 sleep duration were observed (all *p* < 0.05). No significant changes were noted for PSG sleep efficiency, sleep latency, REM latency, WASO, total wake time, or N1 sleep duration in either group (Supplementary Table 2).

Mean change for each outcome was calculated as the delta between post- and pre-intervention values. Changes in objective sleep parameters assessed by polysomnography were generally comparable between the almond and control groups after the 5-month intervention. No significant between-group differences were observed for PSG-derived sleep efficiency, sleep latency, rapid eye movement (REM) latency, wake after sleep onset (WASO), total wake time, or time spent in N1, N2, N3, or REM sleep (all *p* > 0.05). However, a trend toward reduced wake during sleep was observed in the almond group compared with the control group (mean change: −4.78 ± 21.77 vs. 2.73 ± 35.55 min, *p* = 0.094) (Table 2).

**Table 2:** Comparison of mean changes (delta change) in objective and subjective sleep parameters between the almond and control groups.

Characteristics	Almond group (*n* = 88)	Control group (*n* = 87)	*p*
	Mean (SD) (95%CI)		
Objective sleep parameters (polysomnography)			
PSG sleep efficiency (%)	0.63(8.12) (−1.09–2.35)	0.85(7.84) (−0.82–2.53)	0.856
PSG sleep latency (min)	−0.91(37.58) (−7.05–8.87)	−5.34(37.26) (−13.29–2.59)	0.270
REM latency (min)	9.29(102.9) (−12.51–31.10)	−8.7(108.34) (−31.79–14.39)	0.261
PSG WASO (min)	−0.81(30.29) (−7.24–5.61)	1.08(34.76) (−6.33–8.49)	0.702
Wake During Sleep (min)	−4.78(21.77) (−9.38–(−)0.16)	2.73(35.55) (−4.84–10.31)	0.094
Total Wake time (min)	−2.42(41.73) (−11.26–6.42)	−6.68(42.37) (−15.71–2.35)	0.503
N1 (min)	3.13(17.77) (−0.630–6.90)	0.74(23.24) (−4.22–5.69)	0.444
N2 (in min)	−3.89(74.44) (−19.66–11.88)	−22.35(62.13) (−35.59-(−)9.11)	0.077
N3 (in min)	16.07(75.48) (0.08–32.06)	30.08(70.98) (14.96–45.21)	0.208
REM (in min)	−7.31(22.20) (−12.01-(−)2.60)	−5.28(24.25) (−10.45-(−)0.11)	0.565
Subjective sleep parameters (PSQI and ESS)			
Global PSQI score	−3.56 (3.0) (−4.18-(−)2.91)	−2.74(2.83) (−3.34-(−)2.13)	0.068
Sleep efficiency (%)	9.7(13.4) (6.83–12.50)	5.4(10.6) (3.14–7.67)	0.021*
Sleep latency (in min)	−13.6(32.8) (−20.51-(−6.60))	−10.9(38.5) (−19.16-(−)2.74)	0.632
Total sleep duration (min)	0.74(1.36) (0.45–1.03)	0.65(1.33) (0.36–0.93)	0.660
Total ESS score	−2.94(5.03) (−4.01-(−)1.88)	−2.54(4.90) (−3.58-(−)1.50)	0.592

PSG, polysomnography; REM, rapid eye movement; WASO, wake after sleep onset; PSQI, Pittsburgh Sleep Quality Index; ESS, Epworth Sleepiness Scale. Independent *t*-test *p < 0.05 was considered significant.

For subjective sleep outcomes, participants in the almond group demonstrated significantly greater improvements in subjective sleep efficiency, assessed using the Pittsburgh Sleep Quality Index (PSQI), compared with the control group (mean change: 9.7 ± 13.4% vs. 5.4 ± 10.6%, *p* = 0.021). The reduction in global PSQI score also favored the almond group (−3.56 ± 3.0) compared with the control group (−2.74 ± 2.83), although this difference did not reach statistical significance (*p* = 0.068). Changes in sleep latency, total sleep duration, and ESS scores were comparable between groups (all *p* > 0.05) (Table 2).

Further, a repeated-measures two-way ANOVA was conducted to examine the effects of almond consumption on sleep outcomes over the 20-week intervention period. A significant group × time interaction was observed for PSQI-measured sleep efficiency (*p* = 0.021), indicating differential changes in sleep efficiency between the almond and control groups over time. However, the overall between-subjects effect of treatment group was not significant, *F* = 0.25, *p* = 0.621, partial η^2^ = 0.001, suggesting no significant difference in the overall mean outcome between the groups when averaged across time. For the remaining sleep parameters, no significant group × time interactions were observed.

Analysis of covariance (ANCOVA) was performed to compare endline sleep outcomes between the almond and control groups after adjusting for baseline values of each outcome and sex (Table 3). Significant treatment effects were observed for subjective sleep measures assessed using the Pittsburgh Sleep Quality Index (PSQI). Post-intervention, participants in the almond group had significantly lower adjusted global PSQI scores than those in the control group (3.94 ± 0.24 vs. 4.79 ± 0.32; *F* = 6.303, *p* = 0.013, partial *η*^2^ = 0.036), indicating better overall sleep quality. Similarly, adjusted PSQI sleep efficiency was significantly higher in the almond group compared with the control group (94.45 ± 0.72% vs. 91.85 ± 0.42%; *F* = 6.414, *p* = 0.012, partial *η*^2^ = 0.036). Participants receiving almonds also exhibited significantly shorter adjusted PSQI sleep latency than controls (18.46 ± 2.25 vs. 26.29 ± 2.27 min; *F* = 5.92, *p* = 0.016, partial *η*^2^ = 0.033). No significant between-group differences were observed for objective polysomnography (PSG) parameters, including PSG sleep efficiency, PSG sleep latency, REM latency, wake after sleep onset (WASO), total wake time, or time spent in N1, N2, N3, and REM sleep (all *p* > 0.05). Although adjusted wake during sleep was lower in the almond group than in the control group (26.34 ± 2.75 vs. 33.22 ± 2.76 min), the difference did not reach statistical significance (*F* = 3.078, *p* = 0.081, partial *η*^2^ = 0.018).

**Table 3:** Adjusted endline objective and subjective sleep outcomes following the 20-week intervention: analysis of covariance (ANCOVA).

Outcomes	Almond (adjusted mean ± SE)	Control (adjusted mean ± SE)	*F*	*p*-value	Partial *η*^2^
Objective sleep parameters (polysomnography)					
PSG sleep efficiency (%)	85.10 ± 0.74	84.87 ± 0.74	0.049	0.826	0.000
PSG sleep latency (min)	40.02 ± 3.17	35.77 ± 3.18	0.882	0.349	0.005
REM latency (min)	158.17 ± 8.58	158.20 ± 8.63	0.000	0.998	0.000
PSG WASO (min)	37.47 ± 3.22	39.52 ± 3.24	0.199	0.656	0.001
Wake during sleep (min)	26.34 ± 2.75	33.22 ± 2.76	3.078	0.081	0.018
Total wake time (min)	75.17 ± 3.91	72.72 ± 3.94	0.191	0.663	0.001
N1 (min)	13.37 ± 2.14	13.65 ± 2.15	0.009	0.926	0.000
N2 (in min)	202.91 ± 5.50	193.79 ± 5.54	1.339	0.249	0.008
N3 (in min)	169.33 ± 6.65	170.82 ± 6.69	0.024	0.876	0.000
REM (in min)	37.30 ± 2.14	36.43 ± 2.15	0.081	0.776	0.000
Subjective sleep parameters (PSQI and ESS)					
Global PSQI score	3.94 ± 0.24	4.79 ± 0.32	6.303	0.013	0.036
Sleep efficiency (%)	94.45 ± 0.72	91.85 ± 0.42	6.414	0.012	0.036
Sleep latency (in min)	18.46 ± 2.25	26.29 ± 2.27	5.92	0.016	0.033
Total sleep duration (min)	6.69 ± 0.12	6.64 ± 0.12	0.075	0.785	0.000
Total ESS score	6.56 ± 0.46	7.65 ± 0.46	2.809	0.096	0.016

### 3.3 Changes in biochemical markers and anthropometric measurements following the 5-month intervention

Melatonin levels increased more in the almond group (delta change: 87.14 ± 164.5 vs. 45.75 ± 153.74 pg./mL), although this difference did not reach statistical significance (*p* = 0.087). Similarly, IL-6, fasting insulin and HOMA-IR decreased in the almond group though the change was not statistically significant (Table 4). No statistically significant differences were observed between groups for changes in biochemical markers, like vitamin E, serotonin, BDNF, TNF-α, cortisol, fasting glucose and HbA1c (*p* > 0.05 for all).

**Table 4:** Comparison of mean changes (delta change) in biochemical and anthropometric parameters between the almond and control groups.

Characteristics	Almond group (*n* = 88)	Control group (*n* = 87)	*P*
	Mean (SD) (95%CI)		
Biochemical measurements			
Vitamin E (mcg/ml)	0.55(2.80) (−0.04–1.14)	0.37(2.94) (−0.25–1.00)	0.685
Melatonin (pg/mL)	87.14(164.5) (52.28–122.00)	45.75(153.74) (12.98–78.51)	0.087
Serotonin (ng/mL)	−81.48(266.85) (−138.02-(−)24.94)	−84.44(242.08) (−136.03–(−)32.84)	0.939
BDNF (pg/mL)	−23.63(95.91) (−43.95-(−)3.31)	−8.14(76.41) (−24.43–8.14)	0.240
TNF-alpha (pg/mL)	−26.92(130.10) (−54.50–0.65)	−31.07(129.85) (−58.75-(−)3.40)	0.833
IL-6 (pg/mL)	−1.21(13.06) (−3.98–1.55)	−0.29(16.98) (−3.91–3.32)	0.687
Cortisol	0.15(42.07) (−8.76–9.07)	0.68(47.15) (−9.37–10.73)	0.938
Fasting glucose (mg/dL)	1.17(13.82) (−1.76–4.10)	3.11(14.85) (−0.05–6.28)	0.370
Fasting insulin (μU/ml)	−0.25(7.09) (−1.75–1.25)	0.98(4.26) (0.08–1.89)	0.165
HbA1c	0.03(0.47) (−0.07–0.13)	0.01(0.35) (−0.07–0.08)	0.705
HOMA IR	−0.04(1.90) (−0.44–0.36)	0.33(1.12) (0.09–0.57)	0.124
Anthropometric measurements			
BMI (kg/m^2^)	0.09(1.24) (−0.16–0.36)	0.28(0.90) (0.09–0.47)	0.277
Percent body fat	−4.30(3.10) (−5.00-(−)3.65)	−5.23(3.78) (−6.04-(−0)4.43)	0.075
Visceral fat	−0.05(1.59) (−0.39–0.29)	0.60(4.06) (−0.26–1.46)	0.164
Muscle mass (%)	2.32(1.51) (2.00–2.65)	1.93(1.05) (1.71–2.16)	0.050*

BDNF, brain derived neurotropic factor; IL-6, interleukin 6; HbA1C, glycated hemoglobin; HOMA IR, homeostatic model assessment of insulin resistance; Independent *t*-test *p < 0.05 was considered significant.

Changes in BMI, body fat percentage, visceral fat, and muscle mass were similar between groups. The almond group showed a greater increase in muscle mass than the control group (2.32 ± 1.51 vs. 1.93 ± 1.05); however, the between-group difference was not statistically significant (*p* = 0.050) (Table 4).

Analysis of covariance (ANCOVA) was performed to compare endline biochemical and anthropometric outcomes between the almond and control groups after adjusting for baseline values of each outcome and sex (Table 5). Among the biochemical measurements, adjusted melatonin concentrations were higher in the almond group than in the control group (250.63 ± 15.98 vs. 206.19 ± 16.07 pg./mL), with the difference reaching statistical significance (*F* = 3.784, *p* = 0.050, partial *η*^2^ = 0.022). No significant between-group differences were observed for other biochemical parameters. Similarly, no significant treatment effects were observed for any anthropometric outcome after adjustment for baseline values and sex.

**Table 5:** Adjusted endline biochemical measurements and anthropometric measurements following the 20-week intervention: analysis of covariance (ANCOVA).

Outcomes	Almond (adjusted mean ± SE)	Control (adjusted mean ± SE)	*F*	*p*-value	Partial *η*^2^
Biochemical measurements					
Vitamin E (mcg/ml)	3.70 ± 0.24	3.30 ± 0.24	1.368	0.244	0.008
Melatonin (pg/mL)	250.63 ± 15.98	206.19 ± 16.07	3.784	0.050	0.022
Serotonin (ng/mL)	299.11 ± 19.58	308.08 ± 19.69	0.103	0.749	0.001
BDNF (pg/mL)	176.14 ± 7.70	180.86 ± 7.75	0.183	0.669	0.001
TNF-alpha (pg/mL)	103.47 ± 10.93	105.64 ± 11.00	0.019	0.889	0.000
IL-6 (pg/mL)	3.28 ± 1.37	5.32 ± 1.37	1.101	0.295	0.006
Cortisol	115.52 ± 3.99	113.36 ± 4.01	0.144	0.705	0.001
Fasting Glucose (mg/dL)	93.55 ± 1.55	94.75 ± 1.56	0.311	0.578	0.002
Fasting Insulin (μU/ml)	9.57 ± 0.54	10.24 ± 0.54	0.752	0.387	0.004
HbA1c	5.68 ± 0.04	5.67 ± 0.04	0.061	0.806	0.000
HOMA IR	2.25 ± 0.15	2.41 ± 0.15	0.620	0.432	0.004
Anthropometric measurements					
BMI (kg/m^2^)	27.49 ± 0.12	27.64 ± 0.12	0.783	0.378	0.005
Percent body fat	35.63 ± 0.30	35.38 ± 0.30	0.346	0.557	0.002
Visceral fat	10.30 ± 0.33	10.98 ± 0.33	2.060	0.153	0.012
Muscle mass (%)	24.00 ± 0.12	23.84 ± 0.12	0.411	0.522	0.002

### 3.4 Responder analysis

A higher proportion of participants in the almond group achieved a clinically meaningful improvement in sleep quality, defined as a ≥ 3-point reduction in PSQI score, compared with the control group (62.5%, *n* = 55 vs. 54.0%, *n* = 47) (Table 6).

**Table 6:** Mean delta changes in PSG measured sleep parameters in participants with ≥3-point reduction in PSQI score in almonds and control group.

Characteristics	Almond group (*n* = 55)	Control group (*n* = 47)	*p*
	Mean (SD) (95%CI)		
Objective sleep parameters (polysomnography)			
PSG sleep efficiency (%)	1.55(6.89) (−0.31–3.41)	−0.85(8.38) (−3.31–1.61)	0.116
PSG sleep latency (min)	−3.77(33.03) (−12.70–5.16)	−2.08(43.08) (−14.73–10.56)	0.824
R latency (min)	14.73(113.96) (−16.08–45.54)	−0.65(101.77) (−30.53–29.23)	0.477
PSG WASO (min)	4.32(34.42) (−4.98–13.63)	8.02(36.74) (−2.77–18.1)	0.601
Wake during sleep (min)	−0.38(19.93) (−5.77–5.01)	11.64(37.80) (0.54–22.74)	0.043
Total wake time (min)	−3.48(35.16) (−12.98–6.02)	1.47(45.09) (−11.77–14.71)	0.535
N1 (min)	2.32(11.88) (−0.89–5.54)	2.90(21.09) (−3.30–9.10)	0.862
N2 (in min)	4.69(78.85) (−16.63–26.01)	−15.01(68.56) (−35.15–5.11)	0.185
N3 (in min)	26.04(69.86) (7.15–44.93)	10.10(66.21) (−9.33–29.54)	0.242
R (in min)	−7.08(22.27) (−13.10-(−)1.06)	−6.34(21.35) (−12.61-(−)0.07)	0.865

Among these PSQI responders, analysis of polysomnography-derived sleep parameters revealed a significant reduction in wake during sleep in the almond group compared with the control group (−0.38 ± 19.93 vs. 11.64 ± 37.80 min; *p* = 0.043). This indicates that participants who responded subjectively to the almond intervention also demonstrated objective improvements in nocturnal wakefulness (Table 6).

## 4 Discussion

In this randomized controlled trial, daily almond consumption for 20 weeks resulted in significant improvements in subjective sleep quality among adults with poor sleep quality assessed using PSQI and/or ESS. After adjustment for baseline values and sex, participants in the almond group demonstrated lower global PSQI scores, higher subjective sleep efficiency, and a shorter subjective sleep latency compared with the control group. However, no significant between-group differences were observed for most objective sleep parameters measured using polysomnography (PSG), although a trend toward reduced wake during sleep was evident. These findings suggest that regular almond consumption may contribute to meaningful improvement in perceived sleep quality among adults experiencing poor sleep.

Subjective sleep measures are clinically important because they reflect an individual’s overall sleep experience and are strongly associated with quality of life, daytime functioning, and psychological well-being. The discrepancy observed between subjective improvements and the absence of significant changes in PSG-derived sleep parameters warrants careful consideration. Subjective and objective sleep assessments capture different dimensions of sleep and often show only modest agreement in community-dwelling adults. PSG quantifies sleep architecture over a limited number of nights in a laboratory setting, whereas questionnaires such as the PSQI reflect an individual’s perception of sleep over an extended period. Consequently, improvements in sleep continuity, reductions in nocturnal arousals, or a heightened sense of restorative sleep may be perceived by participants without producing substantial changes in conventional PSG-derived variables.

Although overall PSG outcomes were not significantly altered, the observed trend toward reduced wake during sleep in the almond group is noteworthy. Wake during sleep is an important indicator of sleep fragmentation and continuity, and reductions in nocturnal wakefulness are associated with improved sleep maintenance and better next-day functioning. Notably, among participants who achieved clinically meaningful improvements in PSQI scores, wake during sleep was significantly reduced in the almond group compared with the control group. These findings suggest that subjective improvements may have been accompanied by meaningful enhancements in sleep continuity among individuals who responded most favorably to the intervention.

The observed improvements in subjective sleep quality may be explained by the unique nutrient profile of almonds. Almonds contain melatonin, magnesium, unsaturated fatty acids, dietary fiber, and plant protein, all of which have been implicated in sleep regulation (16, 17, 32). Magnesium has been associated with improved sleep quality through its role in regulating neurotransmitter activity and maintaining normal circadian rhythms (33). Almonds also provide tryptophan, an essential amino acid that serves as a precursor for serotonin and melatonin synthesis (16, 17). Increased availability of these sleep-related compounds may contribute to improved sleep perception and overall sleep quality (34). In addition, almonds are rich in vitamin E and other bioactive compounds with antioxidant properties that may help reduce oxidative stress, a factor linked to sleep disturbances (35). The biochemical findings provide additional insights into potential mechanisms underlying the observed effects. The significantly higher adjusted melatonin concentrations observed in the almond group support the biological plausibility of the intervention. Although circulating melatonin levels influenced by numerous physiological and environmental factors, the increase observed following almond consumption suggests that regular intake may enhance endogenous melatonin availability or help preserve circulating concentrations. Almonds are among the few plant foods known to naturally contain melatonin, and previous research has suggested that dietary melatonin may contribute to improved sleep regulation (21, 36). The trend toward higher circulating melatonin levels is therefore biologically plausible and may partly explain the improvements in subjective sleep outcomes. Nevertheless, the relatively small effect size indicates that melatonin alone is unlikely to account for the observed benefits, suggesting that multiple nutritional components of almonds may act synergistically. Future studies incorporating larger sample sizes and repeated hormonal assessments may help clarify the contribution of melatonin to these effects.

Inflammation has emerged as an important contributor to sleep disturbances, with elevated inflammatory cytokines being associated with impaired sleep quality and sleep fragmentation (37, 38). In the present study, reductions in IL-6 and TNF-α were observed in the almond group, although these changes did not reach statistical significance. Almonds contain several constituents with anti-inflammatory properties, including monounsaturated fatty acids, polyphenols, magnesium, and vitamin E. Previous studies have demonstrated beneficial effects of nut consumption on inflammatory status and oxidative stress (39, 40). While our findings do not provide definitive evidence linking anti-inflammatory effects of almonds to improvements in sleep, the favorable trends observed warrant further investigation in studies specifically designed and powered to evaluate inflammatory pathways.

Similarly, improvements in fasting insulin and HOMA-IR were observed in the almond group, although between-group differences were not statistically significant. Sleep quality and metabolic health are closely interconnected, and insulin resistance has been associated with poor sleep and disrupted circadian regulation (41). Almond consumption has previously been shown to improve glycemic control and insulin sensitivity in some populations (42). Therefore, the trend observed in the present study may indicate potential metabolic benefits that could indirectly contribute to improved sleep over longer intervention periods. However, the lack of statistically significant between-group differences suggests that any metabolic effects were modest within the timeframe of the current intervention.

No significant differences were observed between-groups for serotonin, BDNF, cortisol, fasting glucose, HbA1c, or vitamin E. Although serum alpha-tocopherol concentrations increased in the almond group and provided objective evidence of compliance, the magnitude of change may not have been sufficient to directly influence sleep-related outcomes directly. Likewise, the absence of significant changes in cortisol suggests that the intervention did not substantially alter hypothalamic–pituitary–adrenal axis activity. Collectively, these findings indicate that improvements in sleep quality may have arisen through multiple interrelated physiological pathways rather than a single dominant biochemical mechanism.

Anthropometric outcomes were largely unchanged between groups. Changes in BMI, body fat percentage, visceral fat, and muscle mass were comparable between interventions. Although the almond group demonstrated a greater increase in muscle mass, this difference did not reach statistical significance. These findings are consistent with previous evidence indicating that regular nut consumption does not necessarily promote weight gain despite the high energy density of nuts and may support favorable body composition when incorporated into a balanced diet (38, 43). Given that meaningful changes in body composition typically require longer intervention periods and/or concurrent exercise interventions, the limited anthropometric effects observed in this study were not unexpected.

Several strengths enhance the validity of the present findings. These include the randomized controlled design, relatively long intervention duration, objective assessment of sleep using polysomnography, evaluation of both subjective and objective sleep outcomes, comprehensive biochemical assessment, and high adherence rates supported by biochemical compliance markers. The combined use of questionnaire-based and PSG-based assessments provides a more comprehensive evaluation of the effects of almond consumption on sleep. To our knowledge, this is among the first community-based trials to examine the effects of daily almond consumption on sleep architecture using PSG. Previous studies have largely been limited to smaller samples or specific populations, such as university students or individuals with Alzheimer’s disease, and none have evaluated daily almond consumption in otherwise healthy adults (23, 44). An additional methodological strength is that all PSG assessments were conducted on weekdays at both baseline and follow-up. This approach minimized variability attributable to weekday–weekend differences in sleep schedule, social obligations, compensatory sleep, and accumulated sleep debt, thereby improving the comparability of objective sleep measurements across study visits. Dietary intake was assessed throughout the study, participants following special diets were excluded, and all participants were instructed to maintain their habitual dietary patterns. Individuals with excessive alcohol consumption were excluded, while those who consumed alcohol occasionally were advised to avoid alcohol during the intervention. Participants were also instructed to avoid excessive caffeine intake. Physical activity, screen time, and sleep hygiene practices were assessed, and participants were generally within the sedentary to light moderately active physical activity range, reducing the likelihood of confounding due to major differences in activity levels. However, although these lifestyle factors were assessed, detailed data on screen time and sleep hygiene were not included in the present manuscript because they were not primary study outcomes. In addition, chronotype were not systematically monitored during the intervention. Therefore, the potential influence of these factors on sleep outcomes cannot be completely excluded.

Sleep was assessed using a single-night PSG at each time point, which may not fully account for night-to-night variability and may be susceptible to first-night effects. Although compliance with the intervention was excellent, dietary intake outside the intervention was assessed through self-report and residual dietary confounding cannot be excluded. The study included adults with poor sleep quality but excluded individuals with severe obstructive sleep apnea and major psychiatric disorders; consequently, the findings may not be generalizable to clinical sleep-disorder populations. Finally, although statistically significant improvements were observed in subjective sleep outcomes, the effect sizes were modest. Larger multicenter trials are needed to confirm these findings, determine their clinical significance, and further elucidate the underlying biological mechanisms.

## 5 Conclusion

Daily almond consumption for 20 weeks improved subjective sleep quality, sleep efficiency, and sleep latency in adults with poor sleep quality without adversely affecting body weight or metabolic health. Although objective PSG-derived sleep parameters were unchanged, higher circulating melatonin concentrations and reduced nocturnal wakefulness among participants who experienced clinically meaningful improvements in sleep quality provide plausible biological support for the observed benefits. These findings suggest that incorporating almonds into the habitual diet may represent a practical, safe, and sustainable nutritional approach for improving perceived sleep quality. Further studies are needed to confirm the effects of almond consumption on objective sleep physiology and to clarify the biological pathways involved. Given their nutritional value, safety profile, and ease of incorporation into everyday diets, almonds may be considered as a component of broader lifestyle-based strategies aimed at improving sleep health.

## Data Availability

The original contributions presented in the study are included in the article/Supplementary material, further inquiries can be directed to the corresponding author.
