# Supplementary material for: Effects of almond consumption on sleep quality in adults: a randomized controlled trial
Source: Front Nutr. 2026 Jul 27;13:1892442. doi: 10.3389/fnut.2026.1892442 (PMC13454318; doi:10.3389/fnut.2026.1892442)
Supplement: Supplementary file 1 [file Data_Sheet_1.PDF]

Supplementary Table 1: Special Biomarkers Assay Detection Limits, Measurement Ranges, And Intra-Assay And Inter-Assay Coefficients Of Variation

Assay	Catalogue No	Detection Limit (Sensitivity) [Detection Range]	Intraassay/interassay CV
**ST/5-HT(Serotonin/5-Hydroxytryptamine) ELISA Kit**	E-EL-0033	9.38 ng/mL [15.63-1000 ng/mL]	<10%
**Brain-Derived Neurotrophic Factor (BDNF) ELISA**	E-EL-H0010	18.75 pg/mL [31.25-2000 pg/mL]	<10%
**Human TNF-α(Tumor Necrosis Factor Alpha) ELISA Kit**	E-EL-H0109	4.69 pg/mL [7.81-500 pg/mL]	< 10%
**Human MT(Melatonin) ELISA Kit**	E-EL-H2016	9.38 pg/mL [15.63-1000 pg/mL]	<10%
**Human IL-6(Interleukin 6) ELISA Kit**	E-EL-H6156	0.94 pg/mL [1.56-100 pg/mL]	<10%

Supplementary Table 2: Mean Changes in Objective and Subjective Sleep Parameters Following 20 Weeks of Intervention in the Almonds and Control Groups

Characteristics	Almond group (n=88) Mean (SD)			Control Group (n=87) Mean (SD)		
	Pre (Week 0)	Post (Week 20)	P	Pre (Week 0)	Post (Week 20)	p
**OBJECTIVE SLEEP PARAMETERS (Polysomnography)**						
**PSG Sleep Efficiency**	84.6(7.1)	85.2(7.0)	0.466	83.9(5.5)	84.8(7.4)	0.313
**PSG Sleep Latency**	38.5(28.5)	39.4(30.2)	0.821	41.8(24.8))	36.4(29.1)	0.184
**R Latency**	147.4(76.2)	156.7(81.5)	0.399	168.4(85.1)	159.7(78.6)	0.456
**PSG WASO**	38.24(26.71)	37.43(32.14)	0.802	38.48(21.80)	39.56(31.56)	0.773
**Wake During Sleep**	31.46(28.16)	26.69(22.52)	0.043	30.13(20.41)	32.86(30.70)	0.475
**Total Wake time (min)**	76.71(38.11)	74.29(39.75)	0.588	80.29(32.15)	73.61(37.96)	0.145
**N1 (min)**	9.21(11.29)	12.34(21.17)	0.102	13.96(21.30)	14.69(28.85)	0.769
**N2 (in min)**	205.8(55.56)	201.95(51.11)	0.625	217.11(57.65)	194.76(52.83)	0.001
**N3 (in min)**	154.30(62.35)	170.37(72.15)	0.049	139.68(69.28)	169.77(64.86)	0.000
**REM (in min)**	45.87(25.05)	38.57(26.48)	0.003	40.43(23.52)	35.15(19.36)	0.045
**SUBJECTIVE SLEEP PARAMETERS (PSQI &ESS)**						

**Global PSQI score**	7.44 (2.75)	3.90(1.97)	0.000	7.57 (2.74)	4.84(2.71)	0.000
**Sleep Efficiency (%)**	84.8(12.2)	94.5(4.7)	0.000	86.4(10.5)	91.8(8.4)	0.001
**Sleep Latency (in min)**	31.3(31.9)	17.8(14.1)	0.000	37.9(38.5)	27.0(27.5)	0.000
**Total Sleep duration**	5.96(1.25)	6.70(1.14)	0.000	5.98(1.16)	6.63(1.24)	0.000
**Total ESS Score**	9.28(3.91)	6.34(3.91)	0.000	10.41(4.55)	7.87(5.02)	0.000

PSG, Polysomnography. REM, Rapid Eye Movement. WASO, wake after Sleep Onset. PSQI, Pittsburgh Sleep Quality Index. ESS, Epworth Sleepiness Scale.
Paired t-test * $p < 0.05$ was considered significant
